# Assessment of the Mechanical Properties of Sisal Fiber-Reinforced Silty Clay Using Triaxial Shear Tests

**DOI:** 10.1155/2014/436231

**Published:** 2014-04-10

**Authors:** Yankai Wu, Yanbin Li, Bin Niu

**Affiliations:** Civil Engineering and Architecture College, Shandong University of Science and Technology, Qingdao 266590, China

## Abstract

Fiber reinforcement is widely used in construction engineering to improve the mechanical properties of soil because it increases the soil's strength and improves the soil's mechanical properties. However, the mechanical properties of fiber-reinforced soils remain controversial. The present study investigated the mechanical properties of silty clay reinforced with discrete, randomly distributed sisal fibers using triaxial shear tests. The sisal fibers were cut to different lengths, randomly mixed with silty clay in varying percentages, and compacted to the maximum dry density at the optimum moisture content. The results indicate that with a fiber length of 10 mm and content of 1.0%, sisal fiber-reinforced silty clay is 20% stronger than nonreinforced silty clay. The fiber-reinforced silty clay exhibited crack fracture and surface shear fracture failure modes, implying that sisal fiber is a good earth reinforcement material with potential applications in civil engineering, dam foundation, roadbed engineering, and ground treatment.

## 1. Introduction

Fiber-reinforced soil, in which soil is mixed with an appropriate amount of fiber, has many applications in earthwork construction, such as soil modification, roadway embankment reinforcement [1], and earth slopes [2].

At present, many studies have been conducted to experimentally determine the mechanical properties of fiber-reinforced soil. Li et al. reported that the addition of synthetic fibers to cohesive soil could significantly improve the soil's shear strength and enhance the tensile strength of the soil body [3]. Pan and Wang analyzed the feasibility of applying earthwork synthetic fiber to expansive soil and proposed a method for calculating the foundation reinforcement depth and amount of synthetic fiber required to control the soil's swelling deformation [4]. Boominathan and Hari mixed fly ash with polypropylene fibers and performed a series of tests to determine the conditions under which the fiber content, loading, and confining pressures varied with respect to one another [5]. The results indicated that polypropylene could significantly improve the liquefaction strength of the fiber-reinforced soil. Tang et al. investigated the reinforcement of soft soils using synthetic fibers and found that fiber reinforcement restricted the displacement and deformation of soil particles and enhanced the strength of limestone soil and soil cement [6]. Shi et al. performed triaxial tests on lime soil mixed with polypropylene fibers and found that the strength of polypropylene fiber-reinforced lime soil increased with increasing axial strain [7]. As illustrated by the aforementioned studies, many achievements have been made in the study of synthetic fiber-reinforced soil.

However, due to the rising cost of synthetic fibers and energy concerns, renewable plant fibers, such as coir fiber and sisal fiber, have also been considered for soil reinforcement. Santhi and Sayida studied the properties of sisal fiber-reinforced black cotton soil and found that the addition of new fibers decreased the maximum dry density and optimum water content of the soil but improved both the California bearing ratio and unconfined compressive strength [8]. In his academic dissertation, Gaw noted that the use of coir fiber to reinforce soil was effective and practical for improving a soil's ground-bearing capacity and slope stability [9]. Bao and Meng studied sisal fiber-reinforced concrete and proposed an empirical formula for the compression and bending of sisal fiber-reinforced concrete at different ages and with different fiber contents [10]. Many previous researchers have made important achievements regarding plant fiber-reinforced soils. However, there is still no unified theoretical system for describing the mechanical properties of plant fiber-reinforced soils. Although plant fiber has been reported to reinforce black cotton soil and concrete, there are no studies regarding the use of plant fibers to reinforce other types of soils, such as clay, silty clay, and sand.

Based on the aforementioned discussion, we analyzed the mechanical properties of sisal fiber-reinforced soils, determined the optimal percentage of fiber addition to silty clay in terms of strength characteristics, and studied the soil's shear failure modes. This study focuses on the effects of different fiber lengths and contents on the engineering properties of silty clay.

## 2. Materials and Methods

### 2.1. Laboratory Equipment and Materials

**Figure 1:**
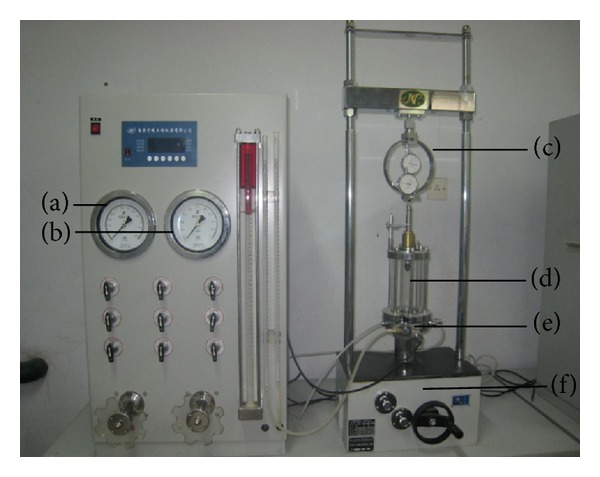
Triaxial shear test apparatus: (a) confining pressure gauge, (b) back-pressure table gauge, (c) proving ring, (d) pressure chamber, (e) pore water pressure sensor, and (f) testing machine.

A TSI-3 automatic strain-controlled triaxial shear apparatus from the Nanjing Soil Instrument Co., Ltd. (China) was used for the tests. This apparatus, which consists of a pore water pressure measurement system, testing machine, pressure chamber, back-pressure control system, and confining pressure control system, can measure the shear strength, deformation characteristics, and pore water pressure during testing. The apparatus can be used to perform unconsolidated undrained shear tests, consolidated undrained shear tests, and consolidated drained shear tests. The strain-control mode of the apparatus is controlled by a single-chip microcomputer, and a stepless variable speed can be realized. The confining pressure and back pressure are controlled automatically by the single-chip microcomputer. The advantages of this apparatus include its ease of operation and compact structure (Figure 1).

This study considered a type of silty clay common to Qingdao, China, which is also very commonly used in engineering applications. Its liquid limit was 30, its plastic limit was 17, and its plasticity index was 13. Before the test, the soil body was sieved through a 1 mm pore sieve and then parched for reservation. Sisal is a leaf fiber with high strength and good toughness; it has been widely used in industrial applications. The fiber content in sisal leaves determines their strength and hardness. Sisals growing in different regions and of different ages have different fiber contents. The sisal fiber used to reinforce the silty clay in this study was collected from Guangxi, China. Its chemical composition was analyzed to evaluate the fiber content in sisal from this region. Conventional procedures were used for this purpose [11]. The results from the chemical composition analysis are provided in Table 1. The sisal fibers that were mixed with the silty clay had an average diameter of 0.25 mm and an average tensile strength of 526 MPa, and cellulose was its main component.

### 2.2. Test Preparation: Compaction Test

A standard indoor compaction test that measured the properties of the sieved soil before it was mixed with sisal fibers was used to determine the relationship between the dry density and water content of the soil samples, which could then be used to determine the soil's maximum dry density and optimum water content. The procedures were performed according to the standards of the Rules of Geotechnical Testing [11].

The compaction test was conducted as follows. First, a group of at least five test samples was produced using the sieved, dried silty clay; the water content of each test sample differed from that of the other samples by 2%. Two of the test samples had a water content greater than the plastic limit, two had a water content below the plastic limit, and one had a water content near the plastic limit. Second, each type of test sample was poured into the compaction canister in three layers, and each layer was compacted 25 times. The height of each soil layer remained fairly constant. Before compaction, the surface of the soil layers was made uniform. During compaction, the compaction hammer was allowed to fall freely such that the soil layer was compacted evenly. After compaction, the height of the test sample was slightly higher than that of the compaction canister wall. Third, the compaction canister was weighed with and without the test sample, with the difference in the measurements being the weight of the wet test sample. Finally, the dry density of each test sample was calculated via (1a) and (1b).

Consider

(1a) $$\begin{array}{c} \rho =\frac{m}{v}, \end{array}$$

(1b) $$\begin{array}{c} \rho_{d}=\frac{\rho }{1+0.01w}, \end{array}$$

where *ρ*, *m*, *v*, *w*, and *ρ*_*d*_ are the density, weight, volume, water content, and dry density of the tested sample, respectively.

**Figure 2:**
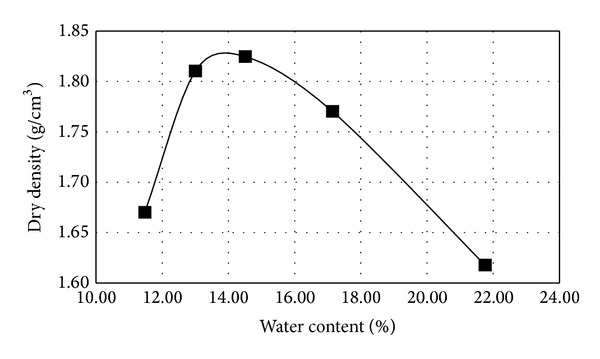
Dry density-water content curve.

This procedure was used to obtain a series of dry densities that correspond to the water content of the test samples and to identify the relationship between the dry density and water content. The relationship between the water content and dry density as determined from the compaction test is shown in Figure 2. The water content corresponding to the maximum dry density is the optimum water content of the material. With the optimal water content, the soil samples can achieve an optimal density, even at the same compaction energy. From Figure 2, the optimum water content of this soil was determined to be 14%.

### 2.3. Sample Preparation

Approximately 5 kg of the dried soil sample was weighed and placed on the plate; then, a certain amount of 0.5 cm long sisal fibers was mixed with the soil. Approximately, 140 mL of distilled water (corresponding to the optimum water content of 14%) was sprayed onto the well-mixed soil using a sprinkler. To ensure that the water, soil, and sisal fibers were uniformly mixed, the well-mixed soil was maintained in a sealed aquarium for 24 h at room temperature (25°C). Then, the mixed soil was divided into three layers to be placed into the light compactor, which has a diameter of 39.1 mm and a height of 80 mm. When the first layer of mixed soil was placed into the compactor, it was compacted 25 times by the compaction hammer. The second and third layers were then successively added and compacted 25 times by the compaction hammer. Therefore, the mixed soil sample had a resulting diameter of 39.1 mm and height of 80 mm. Soil test samples with and without sisal fiber reinforcement with different fiber contents (0.5%, 1.0%, and 1.5%) and fiber lengths (0.5, 1.0, and 1.5 cm) were created.

Triaxial shear tests were performed to study the changes in the mechanical properties of sisal fiber-reinforced soil resulting from variations in the fiber length and content. According to a previous study [12], the mechanical properties of fiber-reinforced soil are optimal at fiber contents of 1-2%. Thus, during the triaxial shear test, the original 1 to 2 m long sisal fibers were cut to lengths of 5, 10, and 15 mm. The cut pieces of the sisal fibers were placed in a 10% NaOH solution for 5 min for pretreatment; pretreatment swells the cellulose, decreases the cellulose crystallinity index, and facilitates the hydrolyzation of lignocellulose by enzymes. Then, the sisal fibers were removed from the 10% NaOH solution and dried. The sisal fibers of different lengths details of the test soil samples are provided in Table 2. The triaxial shear test samples were created according to the optimum water content determined from the compaction tests.

**Figure 3:**
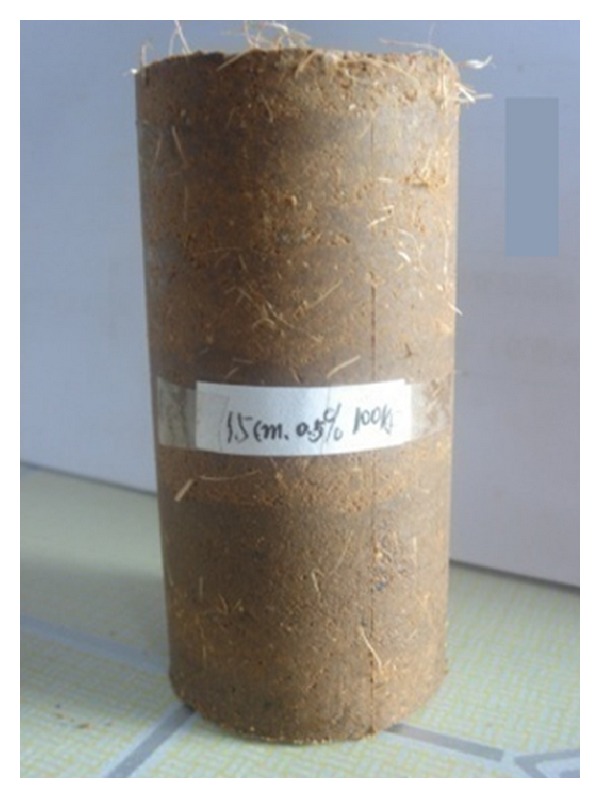
Sisal fiber-reinforced soil sample (fiber length of 15 mm and content of 0.5%).

Four triaxial shear test samples were tested for each combination of fiber length and content. Triaxial shear tests were conducted on the 10 sisal fiber-reinforced soil sample groups. Thus, 40 test samples were prepared. Unconsolidated undrained triaxial shear tests were performed separately for each test sample under the condition that the soil body would not compact and drain under confining pressures of 100, 200, 300, and 400 kPa.  Figure 3 presents a photograph of a sisal fiber-reinforced soil sample.

### 2.4. Triaxial Shear Tests

After the samples were prepared, triaxial shear tests were performed in accordance with the standards of the Rules of Geotechnical Testing [11].

#### 2.4.1. Nonreinforced Soil

**Figure 4:**
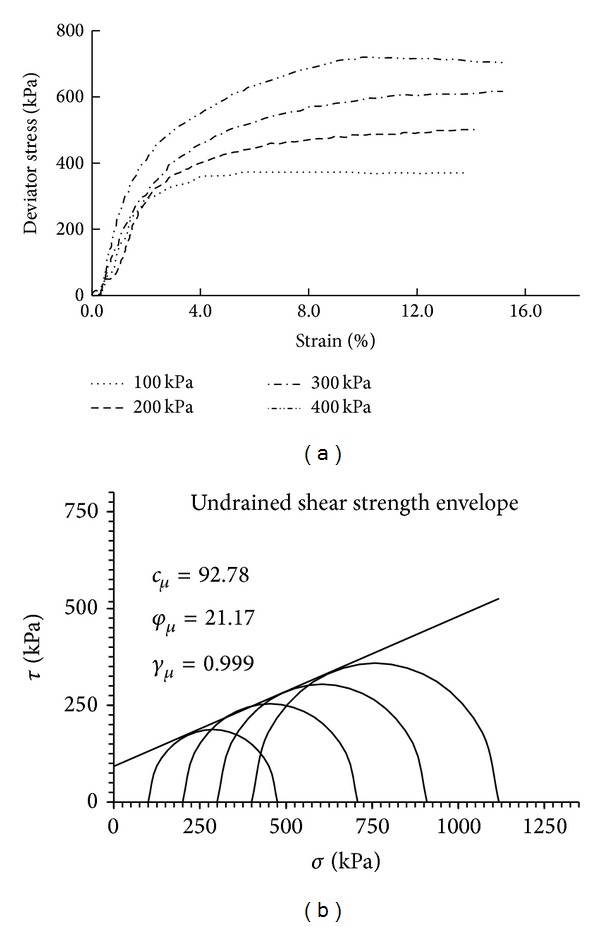
Results of a triaxial shear test on a nonreinforced soil sample: (a) strain versus stress curve and (b) strength envelope.

Unconsolidated undrained triaxial shear tests were conducted on the nonreinforced soil. Four nonreinforced silty clay samples were sheared under confining pressures of 100, 200, 300, and 400 kPa.  Figure 4(a) presents the strain-deviator stress curves at the different confining pressures. Mohr-Coulomb strength theory, as shown in Figure 4(b), indicates that the cohesion (92.78 kPa) and friction angle (21.17°) of the silty clay without fiber were achieved. The test results did not exhibit any clear peaks in the strain-deviator stress curves, indicating the occurrence of strain softening.

#### 2.4.2. Soil Mixed with 5 mm Long Sisal Fibers

**Figure 5:**
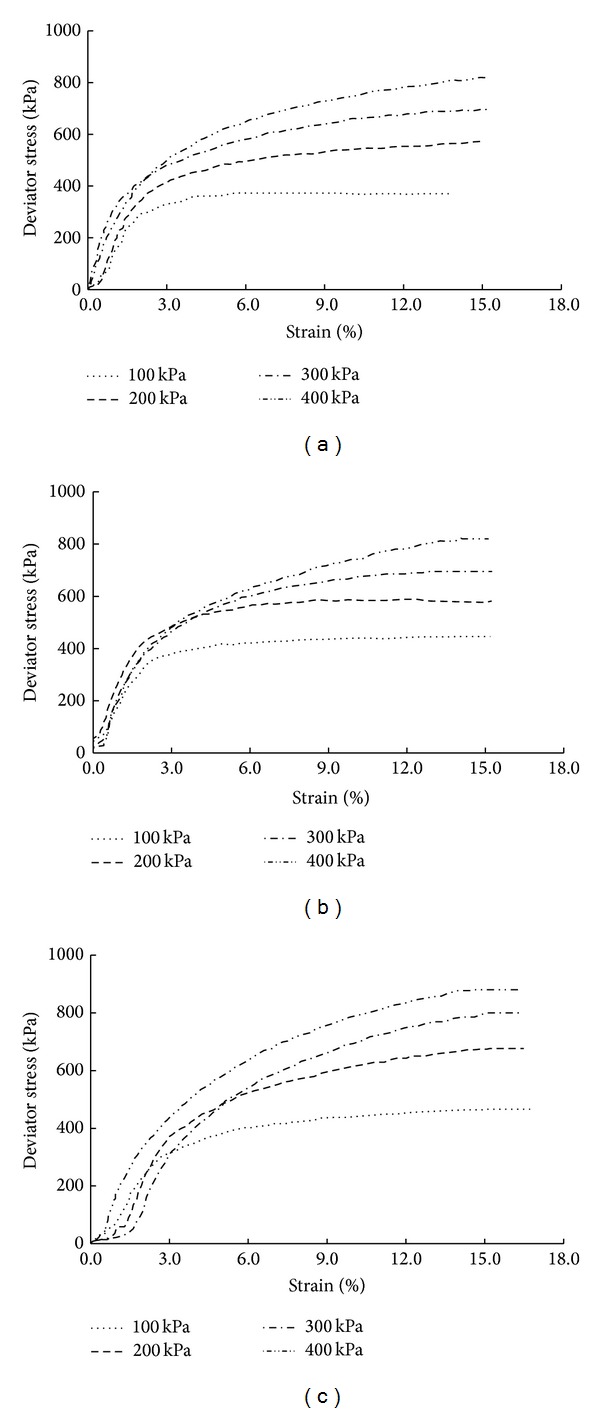
Stress-strain curves of soil reinforced with 5 mm long sisal fibers: (a) sisal fiber content of 0.5%, (b) sisal fiber content of 1.0%, and (c) sisal fiber content of 1.5%.

Triaxial shear tests were conducted on soil mixed with 5 mm long sisal fibers under confining pressures of 100, 200, 300, and 400 kPa.  Figure 5 displays the test results, which indicate that the sisal fiber-reinforced soil exhibits strain hardening with increasing sisal fiber content. The strain hardening phenomenon becomes increasingly clear with increasing confining pressure.

#### 2.4.3. Soil Mixed with 10 mm Long Fibers

**Figure 6:**
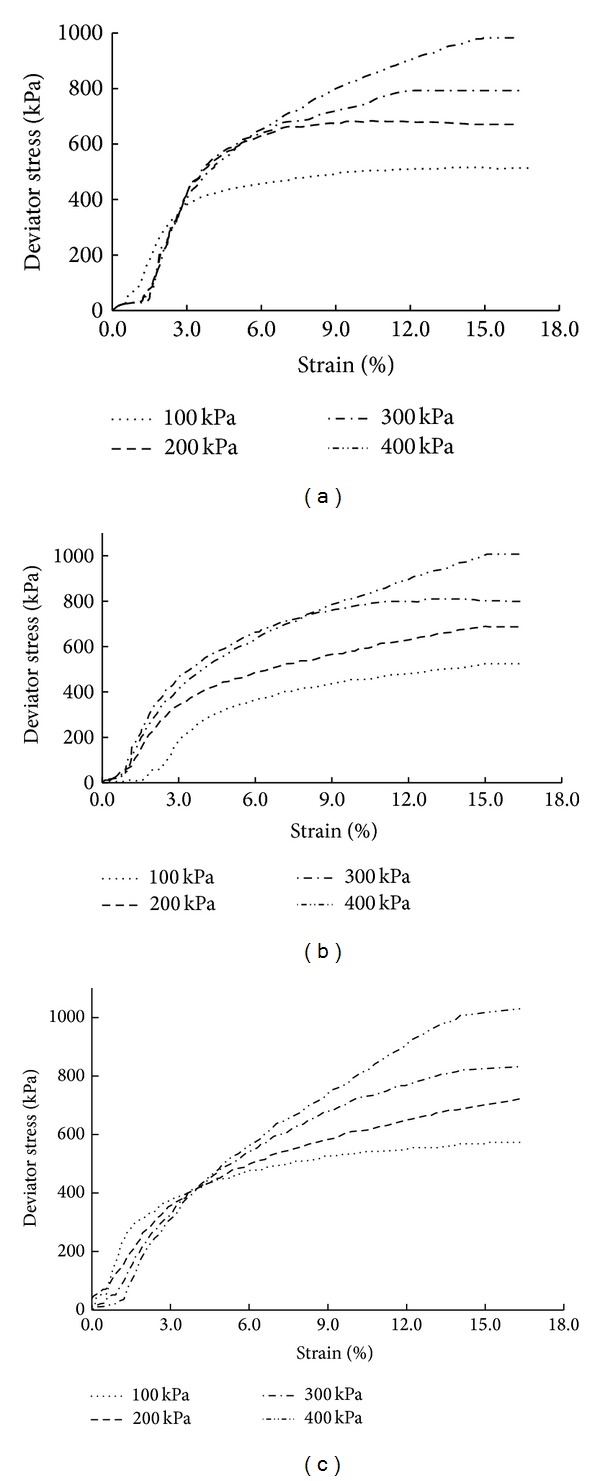
Stress-strain curves of soil reinforced with 10 mm long sisal fibers: (a) sisal fiber content of 0.5%, (b) sisal fiber content of 1.0%, and (c) sisal fiber content of 1.5%.

Triaxial shear tests were conducted on soil mixed with 10 mm long sisal fibers under confining pressures of 100, 200, 300, and 400 kPa.  Figure 6 displays the test results, which indicate that the sisal fiber-reinforced soil exhibits strain hardening with increasing sisal fiber content. For a fiber content of 1.0%, the sisal fiber-reinforced soil exhibits strain hardening for all confining pressures, and no stress peak is observed with increasing strain.

#### 2.4.4. Soil Mixed 15 mm Long Sisal Fibers

**Figure 7:**
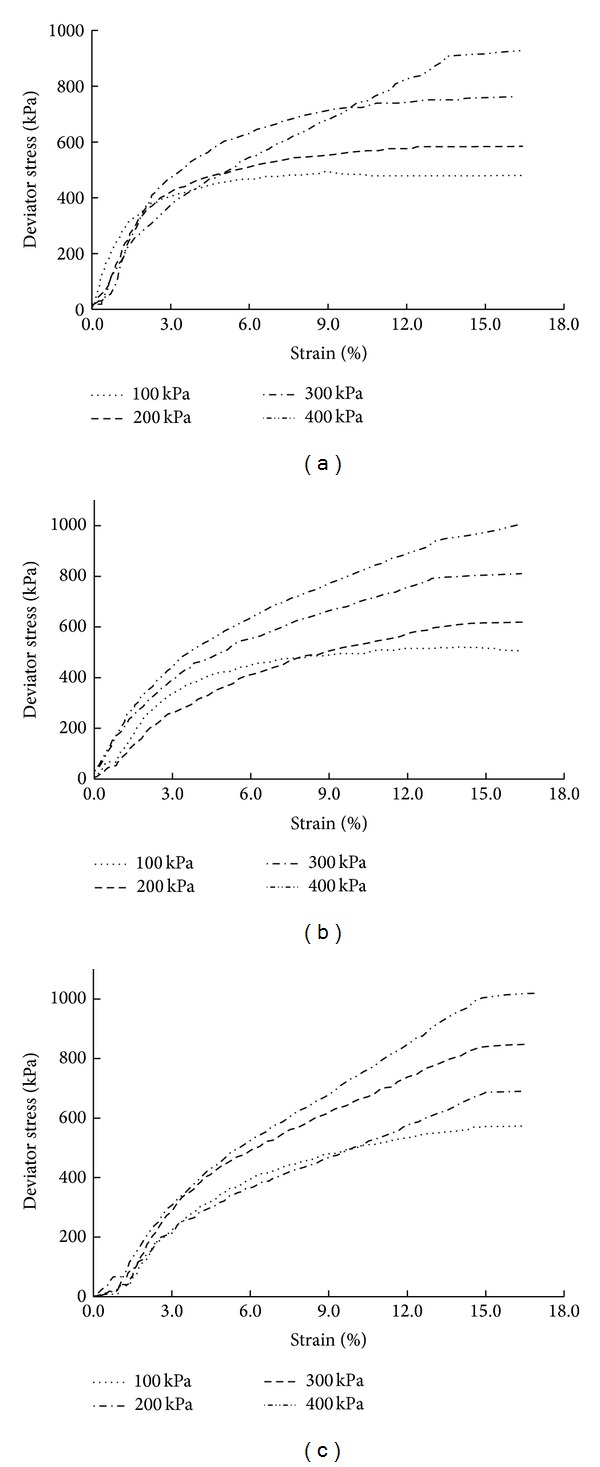
Stress-strain curves of soil reinforced with 15 mm long sisal fibers: (a) sisal fiber content of 0.5%, (b) sisal fiber content of 1.0%, and (c) sisal fiber content of 1.5%.

Triaxial shear tests were conducted on soil mixed with 15 mm long sisal fibers under confining pressures of 100, 200, 300, and 400 kPa.  Figure 7 displays the test results, which indicate that the sisal fiber-reinforced soil exhibits strain hardening for all sisal fiber contents. In particular, the stress clearly increases with increasing strain when the fiber content is 1.0%. There is no clear decrease in stress when the strain exceeds 15%.

## 3. Results and Discussion

### 3.1. Triaxial Shear Test Results

Based on the principle of the triaxial shear test, for the strain-stress curve without the peak strength, we took the corresponding stress for the peak strength to be 15%. The peak deviator stresses of the nonreinforced and sisal fiber-reinforced silty clay soils were calculated from Figures 5–7 according to the stress-strain curves (Table 3). The peak strength of the sisal fiber-reinforced soil increased with increasing fiber length and content. The peak strength increase of the sisal fiber-reinforced soil could not be clearly observed for fiber lengths exceeding 10 mm.

**Figure 8:**
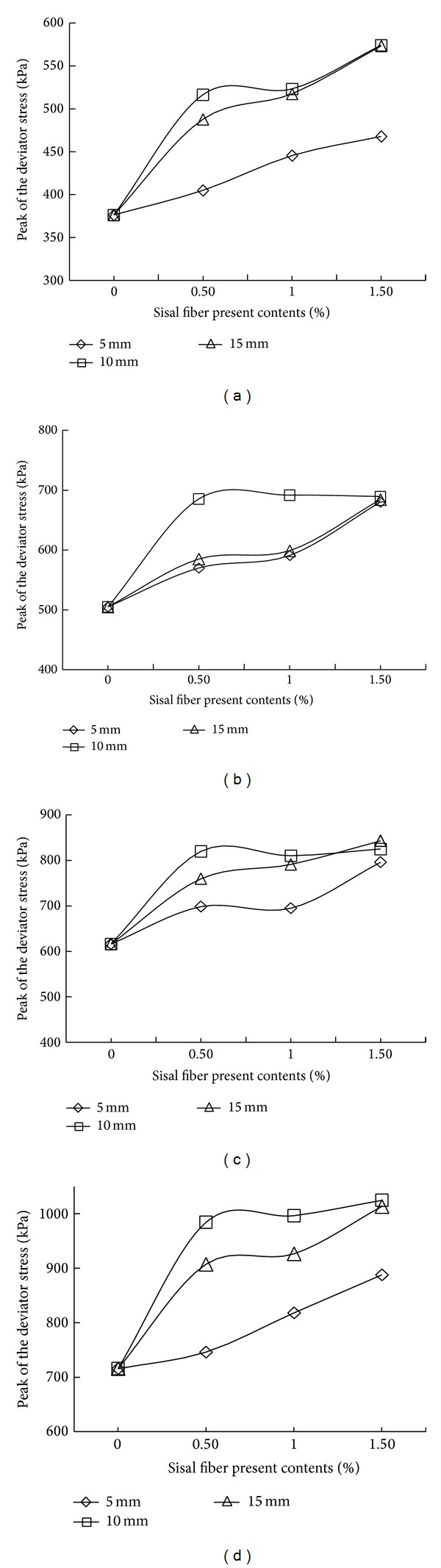
Peak deviator stress versus sisal fiber content: (a) confining pressure of 100 kPa, (b) confining pressure of 200 kPa, (c) confining pressure of 300 kPa, and (d) confining pressure of 400 kPa.

From the data in Table 3, the peak deviator stress versus the sisal fiber content curve was generated under a constant confining pressure to analyze the mechanical properties of the sisal fiber-reinforced soil (Figure 8). As shown in Figures 8(a)–8(d), the peak deviator stress of the soil with a 0.5% fiber content was significantly higher than that of the nonreinforced soil, and the peak deviator stress of the soil with a 1% fiber content was higher than that of the soil with a 0.5% fiber content. Finally, although the peak deviator stress of the soil with a 1.5% fiber content was greater than that of the soil with a fiber content of 1%, this difference was not as pronounced. However, our results were inconsistent with those reported in the literature [8]. According to previous reports, a sisal fiber content of 0.5% and fiber length of 2.5 cm provide the best unconfined compressive strength. The primary reason for these different conclusions is that the black cotton soil studied by Santhi Krishna presumably has a swell-shrink characteristic that differs from that of silty soil.

### 3.2. Cohesion and Internal Friction Angle of the Samples

The mechanical properties, cohesion, and internal friction angle can be obtained from the triaxial shear test results (shown in Figures 4–8), as shown in Table 4. The following conclusions can be drawn from Table 4.

1. The cohesion of the sisal fiber-reinforced soil was significantly greater than that of the nonreinforced soil.
2. Fiber reinforcement did not significantly influence the internal friction angle of the soil, which ranges from approximately 21° to 25°.
3. For a given fiber length, the cohesion of the sisal fiber-reinforced soil increased with increasing fiber content.
4. For a given fiber content, the test samples reinforced with 10 mm long fibers exhibited a significantly higher cohesion than those reinforced with 5 mm long fibers.

Reinforcement with 10 mm long sisal fibers increased the shear strength by approximately 20%, which is only slightly less than that obtained with the 15 mm long fibers. Thus, excessive fiber reinforcement would not be effective for improving the strength of fiber-reinforced soils considering the contact surface of the soil particles and fiber. In contrast, fiber clumping would likely occur due to insufficient mixing between the fiber and soil. Furthermore, an excessive amount of fiber leads to reduced contact among the soil particles of the silty clay, thereby reducing the cohesion of the soil body and increasing the relative ease with which sliding can occur between the soil particles.

Based on these results, a sisal fiber length of 10 mm, rather than 5 mm, should be used to improve the shear strength of soil bodies.

### 3.3. Fracture Characteristics of the Sisal Fiber-Reinforced Soil

The test samples exhibited different fracture modes due to differences in fiber content, fiber length, and confining pressure.

#### 3.3.1. Crack Fracture Mode

**Figure 9:**
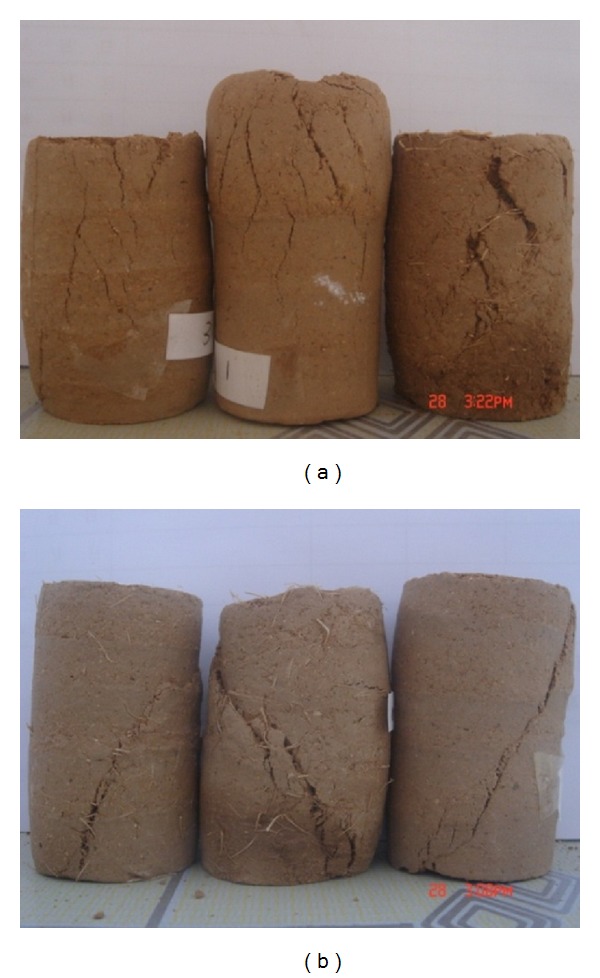
Fracture modes of the fiber-reinforced soil: (a) crack failure mode for soil reinforced with 15 mm long sisal fibers and (b) shear surface failure mode for soil reinforced with 5 and 10 mm long sisal fibers.

As shown in Figure 9(a), the fiber content significantly influences the fracture mode. The crack fracture mode is the prominent facture mode, particularly in the soil reinforced with 15 mm long sisal fibers. When several cracks are present simultaneously, the majority of the cracks are vertical and possess small, irregular inclinations. In most cases, more cracks were observed in the test specimens reinforced with 15 mm long fibers. The fracture surface did not develop along a certain plane as the deviator stress was increased because the 15 mm long fibers could restrain the development of the fracture surface. The crack development increased gradually with increasing fiber content and length.

#### 3.3.2. Surface Shear Fracture Mode

The specimens reinforced with 5 and 10 mm long sisal fibers exhibited a surface shear fracture mode rather than a crack failure mode as the deviator stress was increased. As shown in Figure 9(b), the shear surface appeared abruptly within a short amount of time due to a brittle fracture. Some shearing surfaces extended from the bottom to the top of the test samples, whereas others extended from the bottom to the middle. The development of the fracture surface in the reinforced soil was similar to that in the nonreinforced soil. The angle between the fracture surface and the horizontal plane was 45 + *π*/2. The location of the fracture surface of the sisal fiber-reinforced soil conformed to Mohr-Coulomb strength theory. These results indicate that Mohr-Coulomb strength theory is suitable for evaluating the mechanical properties of the sisal fiber-reinforced soil for fiber lengths of less than 10 mm.

This study has some limitations. The triaxial tests in this study did not consider the influence of water on the mechanical properties of the reinforced soil. The triaxial shear tests were conducted under optimal water content and compaction conditions. However, there is a large difference between the processes of testing and engineering an actual structure. Furthermore, this investigation only conducted triaxial shear tests for silty clay reinforced with sisal fiber. These tests are not sufficiently representative for widespread applications; therefore, the mechanical properties of muddy, sandy, and expansive soils mixed with sisal fibers should be investigated in future studies.

## 4. Conclusions

This study analyzed the influence of fiber length and content on the mechanical properties of sisal fiber-reinforced silty clay.

The silty clay reinforced with 10 mm long fibers can increase the deviator pressure peak and cohesion by approximately 20% compared to the soil reinforced with 5 mm long fibers. However, these two parameters do not continue to increase, and in fact decrease, with further increases in the fiber length. The sisal fiber-reinforced soil exhibits crack fracture and surface shear fracture modes. The factors influencing which type of fracture mode occurs include the fiber content and fiber length. Mohr-Coulomb strength theory is suitable for evaluating the mechanical properties of sisal fiber-reinforced soil when the sisal fiber length does not exceed 10 mm.

The results presented here can provide a design basis for the use of sisal fiber-reinforced soil in engineering as well as the optimum sisal fiber length and content in roadbed, dam foundation, and coastal engineering applications. Future research on sisal fiber-reinforced soil should focus on the durability of plant fiber-reinforced soil and extend the applications of such soils.

## Figures and Tables

**Table 1:** Chemical composition of the sisal fibers used in this study.

Component	%
Cellulose	64.9
Hemicellulose	13.7
Lignin	10.4
Pectin	0.7
Water-soluble matter	1.3
Lipids and waxes	0.2
Water	8.8

**Table 2:** Sisal fiber lengths and contents used in the triaxial shear tests.

Sisal fiber length (mm)	Sisal fiber content (mass percentage)		
0	0		
5	0.5%	1.0%	1.5%
10	0.5%	1.0%	1.5%
15	0.5%	1.0%	1.5%

**Table 3:** Peak deviator stress differences at soil sample failure (kPa).

Confining pressure (kPa)	Content Length	Fiber length of 5 mm			Fiber length of 10 mm			Fiber length of 15 mm		
	General soil	0.5%	1.0%	1.5%	0.5%	1.0%	1.5%	0.5%	1.0%	1.5%
100	376	405	446	468	517	523	574	488	519	573
200	505	570	591	680	685	692	690	585	599	695
300	617	699	695	796	820	810	825	760	791	843
400	716	746	818	888	984	997	1,025	907	927	1,037

**Table 4:** Shear strength results obtained from the triaxial shear tests.

Fiber content	Nonreinforced soil		Fiber length					
			5 mm		10 mm		15 mm	
	*C* (kPa)	*φ* (°)	*C* (kPa)	*φ* (°)	*C* (kPa)	*φ* (°)	*C* (kPa)	*φ* (°)
0.50%	93	21.2	108	22.5	115	22.3	104	24.3
1.00%	93	21.2	111	25.9	114	26.0	113	25.4
1.50%	93	21.2	116	24.7	128	24.7	129	25.2
